# The Efficacy and Tolerability of Radiosurgery in Treating Benign Meningiomas: A Dose Comparison Study from a Single-Center Analysis

**DOI:** 10.3390/life14060664

**Published:** 2024-05-23

**Authors:** Hyun-Jeong Cho, Jong-Min Lee, Sung-Ho Park, Jun-Bum Park, Na-Young Jung

**Affiliations:** 1 Department of Neurosurgery, Ulsan University Hospital, University of Ulsan College of Medicine, Ulsan 44033, Republic of Korea; 0735491@uuh.ulsan.kr (H.-J.C.); jmlee@uuh.ulsan.kr (J.-M.L.); parkjb@uuh.ulsan.kr (J.-B.P.); 2 Department of Neurosurgery, TrueBeam Radiosurgery Center, Ulsan University Hospital, Ulsan 44033, Republic of Korea; michael@uuh.ulsan.kr

**Keywords:** dose, edema, meningioma, stereotactic radiosurgery, toxicity

## Abstract

This retrospective study aimed to evaluate the impact of radiation dose on the outcomes of stereotactic radiosurgery (SRS) for benign meningiomas and determine an optimal dosing strategy for balancing tumor control and treatment-related toxicity. Clinical data of 147 patients with 164 lesions treated between 2014 and 2022 were reviewed. Primary outcomes included progression-free survival (PFS), local control rate (LCR), and radiation-induced toxicity, with secondary outcomes focusing on LCR and radiation-induced peritumoral edema (PTE) in two dose groups (≥14 Gy and <14 Gy). The results revealed a median follow-up duration of 47 months, with 1-year, 2-year, and 5-year PFS rates of 99.3%, 96.7%, and 93.8%, respectively, and an overall LCR of 95.1%. Radiation-induced toxicity was observed in 24.5% of patients, primarily presenting mild symptoms. Notably, no significant difference in LCR was found between the two dose groups (*p* = 0.628), while Group 2 (<14 Gy) exhibited significantly lower PTE (*p* = 0.039). This study concludes that SRS with a radiation dose < 14 Gy demonstrates comparable tumor control with reduced toxicity, advocating consideration of such dosing to achieve a balance between therapeutic efficacy and safety.

## 1. Introduction

Meningioma, a primary intracranial tumor originating from the meninges enveloping the brain and spinal cord, predominantly manifests as a benign neoplasm, with only a few exhibiting malignant characteristics [1,2]. Currently, they account for 40.8% of all primary intracranial neoplasms [3]. Surgical resection remains the primary treatment modality for symptomatic or proliferating meningiomas; however, stereotactic radiosurgery (SRS) has emerged as an effective adjuvant or alternative intervention for low-grade meningiomas. This is particularly relevant for tumors close to critical anatomical structures, recurrent lesions, or cases where resection or general anesthesia poses a high risk to patients [4,5,6].

Numerous studies have substantiated the efficacy of SRS in achieving robust tumor control and preserving neurological function over short and long-term durations [7,8,9,10,11,12,13,14]. In a comprehensive systematic review by Marchetti et al. [9], encompassing findings from the International Stereotactic Radiosurgery Society, single-fraction SRS at a prescribed dose of 12–15 Gy for meningiomas exhibited notable efficacy. The results revealed 10-year local control rates (LCR) of 71–100% and progression-free survival (PFS) rates of 55–97% [9]. Additional investigations corroborated these findings, demonstrating an LCR of 87–100%, particularly when the administered dose was within the range of 12–16 Gy. Notably, a 10-year LCR > 90% has been consistently observed in World Health Organization grade I meningiomas [8,15].

SRS is extensively used in treating meningiomas; however, a degree of uncertainty persists regarding the optimal radiation dosage, enduring implications on lesion control, and the potential for radiation-induced complications. A universally accepted guideline for dose selection is absent, compelling practitioners to rely on the amalgamation of empirical data and institutional experience [15]. Therefore, it is imperative to continually refine our understanding of the most reasonable radiation dosing for meningiomas, necessitating a delicate equilibrium between effective tumor control and the mitigation of treatment-related adverse effects.

The treatment paradigm has undergone a transformative evolution at our institution. Previously, we administered a comparatively elevated dose of ≥14 Gy to patients with meningiomas who underwent SRS. However, guided by our accrued experience and an expanding body of evidence, a deliberate shift in strategy has occurred, leading to a recent reduction in the mean prescribed dose to <14 Gy. This study aimed to comprehensively examine the clinical outcomes and associated toxicities of radiosurgery for meningiomas. The present study investigated parameters such as LCR and radiation-induced peritumoral edema (PTE) using a comparative analysis between the two cohorts subjected to distinct radiation doses. The overarching objective was to provide contemporary insights into the optimal radiation dose, thereby contributing valuable perspectives for informed clinical decision-making.

## 2. Materials and Methods

This retrospective study comprehensively examined the medical records and radiology reports of patients subjected to SRS for benign meningiomas. Diagnosis involved histopathological findings through open resection or identifying characteristic imaging features consistent with benign meningiomas, validated by a consensus between neurosurgeons and neuroradiologists based on magnetic resonance imaging (MRI) observations. We treated 162 patients with meningiomas at our institution using TrueBeam radiosurgery between March 2014 and December 2022. The inclusion criteria were benign meningioma diagnosis and undergoing single-session SRS, either as a primary intervention or as an adjuvant measure. Individuals who underwent fractionated or repeated radiosurgery for identical lesions were excluded. Patients lost to follow-up were also excluded due to the unavailability of treatment outcome data.

Pretreatment high-resolution T1-weighted MRI with a slice thickness of 1 mm and gadolinium enhancement was acquired for treatment planning. In addition, a contrast-enhanced computed tomography (CT) scan, with a slice thickness of 1.5 mm, was conducted with the patient immobilized in a thermoplastic mask using a compatible fiducial localizer. The MRI and CT images were integrated, with subsequent delineation of the target and all organs at risk performed on the MR images, using the iPlan RT Image version 4.1 and iPlan RT Dose version 4.5 planning software (Brainlab, Feldkirchen, Germany). Typically, lesions were subjected to an 85–90% isodose line. Single-isocenter treatment plans were executed for all patients employing several static beams or dynamic conformal arcs with three to five gantry positions. The total dose was determined based on the target pathology, lesion size, previous treatments, and proximity to critical structures. The prescribed dose was delivered to each patient in a single fraction through a Varian TrueBeam STx linear accelerator (Varian Medical Systems, Palo Alto, CA, USA). Clinical examination and imaging follow-up were conducted 6 months after radiosurgery, followed by annual assessments.

The investigated variables included age, sex, meningioma location, prior resection history, histologic subtype, initial target volume, and various irradiation parameters, including prescription dose, conformity index (CI), and coverage. The primary outcome measures for all enrolled patients were LCR, PFS, and radiation-induced toxicity. A secondary analysis compared LCR and PTE between the two groups stratified based on their prescription doses: ≥14 Gy (Group 1) and <14 Gy (Group 2). The period of local tumor control was defined as the time between initial radiosurgery and the date of uncontrolled or recurrent lesion identified on follow-up images. LCR was categorized following the Response Assessment in Neuro-Oncology Working Group criteria as [16] (1) complete response (CR), signifying the total disappearance of the target lesion, (2) partial response (PR), indicating a reduction in the sum of the maximal perpendicular diameters by ≥50% relative to baseline, (3) minor response (MR), denoting a decrease between 25% and 50%, encompassing 25%, (4) stable disease (SD), signifying cases that do not fit other classifications, such as <25% decrease but <25% increase in area relative to nadir, and (5) progressive disease (PD), encompassing an increasing lesion size > 25%. Radiation-induced toxicity was evaluated and categorized according to the Common Terminology Criteria for Adverse Events (CTCAE) version 5.0 [17].

This study adhered to the guidelines stipulated in the Strengthening the Reporting of Observational Studies in Epidemiology statement. All data acquisition and analysis procedures were approved by the Institutional Review Board (IRB number: #2023-10-016), and the need for written informed consent was waived.

All statistical analyses were performed using SPSS version 20 (IBM, Armonk, NY, USA). The primary outcome measures were assessed by computing the LCR, PFS, and radiation-induced toxicity estimates from the date of the initial treatment using the Kaplan–Meier method. The log-rank test was employed for the significant comparisons of LCR and PTE between the two groups in the secondary analysis. Continuous variables were analyzed using the t-test, whereas categorical variables were examined using the chi-square and Fisher’s exact tests. The Cox proportional hazards method was used to identify predictors of LCR and PTE. Factors with a *p*-value < 0.05 in the univariate analysis were entered into a multivariate analysis. Statistical significance was set at *p* < 0.05.

## 3. Results

### 3.1. Demographics

Between March 2014 and December 2022, 162 patients underwent SRS for 190 meningiomas at our hospital. This study enrolled 147 patients with 164 treated lesions following the exclusion of 15 patients with 26 lesions, comprising 8 meningiomas in 8 patients subjected to fractionated SRS, 7 meningiomas in 7 patients lost to follow-up, and 11 repeatedly treated meningiomas.

The mean age of the cohort was 61 years (range: 37–79 years), with 35 males (23.8%) and 112 females (76.2%). Most patients (55.1%) were asymptomatic, whereas the rest presented with diverse symptoms, including headache, dizziness, visual disturbances, nausea, motor weakness, hearing decline, facial pain, facial palsy, tremors, and seizures. Objective neurological manifestations, including hemiparesis, dysesthesia, visual impairment, and cranial nerve deficits, were observed in 13 patients (8.8%). Diagnostic modalities included MRI in 122 patients, whereas 25 patients (17%) with a history of open resection underwent histopathological confirmation. The lesion distribution included 60 skull-base meningiomas (36.6%) and 104 non-skull-base meningiomas (63.4%). The median follow-up duration was 47 months (range: 12–122 months). Table 1 presents a detailed overview of the patients’ clinical characteristics.

### 3.2. Tumor Control

The average target volume for single-session SRS was 4.49 cm^3^ (range: 0.33–13.9 cm^3^), with a median dose of 14 Gy (range: 12–16 Gy). The mean coverage was 99.32% (range: 90–100%), and the mean CI was 1.80 (range: 1–4.62). Dose parameters exhibited a maximum dose of 16.3 Gy (range: 14.2–23.6 Gy), a minimum dose of 12.3 Gy (range: 5.8–15.2 Gy), and a mean dose of 15.5 Gy (range: 12.8–19.2 Gy). The two groups showed no significant differences in the target volume, lesion location distribution, or treatment parameters, except for the radiation dose, as detailed in Table 2.

**Figure 1:**
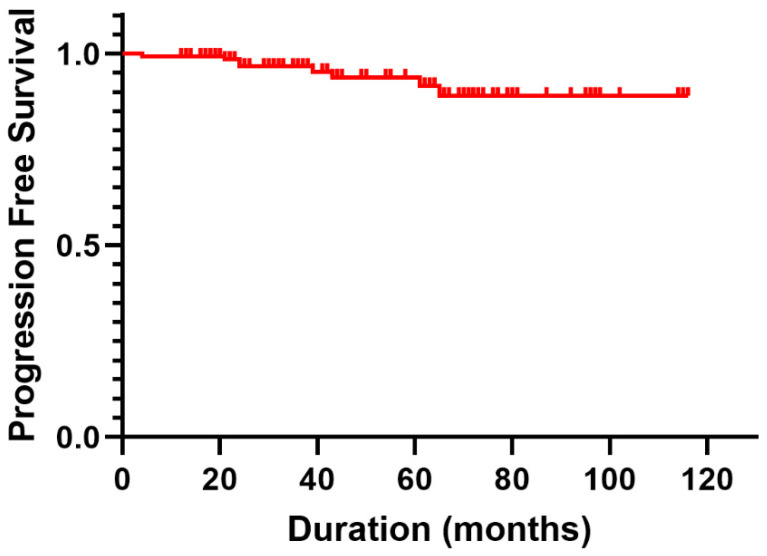
Kaplan–Meier curve showing overall progression-free survival.

During the follow-up period, progression occurred in eight patients (5.4%). Notably, the 1-, 2-, and 5-year PFS rates were 99.3%, 96.7%, and 93.8%, respectively, as illustrated by the Kaplan–Meier curves in Figure 1. Among the eight patients with recurrent meningiomas, three underwent open resection, five underwent repeat SRS, and one who underwent repeat SRS for meningothelial meningioma exhibited malignant progression to atypical meningioma, ultimately requiring resection.

The study cohort’s overall crude LCR was 95.1%. No CR was observed; however, PR was observed in 10 lesions (6.1%), MR in 12 lesions (7.3%), and SD in 134 lesions (81.7%). Only eight lesions (4.9%) displayed signs of PD, necessitating additional SRS or resection, as summarized in Table 3. The LCR over different time intervals was estimated as 99.4%, 97.0%, and 94.5% at 1-, 2-, and 5-year follow-ups, respectively. When comparing the LCR between the two groups (Group 1 and Group 2), with four cases of PD in each group, no significant difference was observed (*p* = 0.628). The specific LCRs for each group at different time points were as follows: 1-year LCR (Group 1: 98.4% vs. Group 2: 100.0%), 2-year LCR (Group 1: 96.6% vs. Group 2: 97.2%), and 5-year LCR (Group 1: 94.7% vs. Group 2: 93.6%). Univariate and multivariate analyses demonstrated that prior surgery and tumor volume > 10 cm^3^ were significantly related to local tumor control (Table 4).

### 3.3. Radiation-Induced Toxicity

Radiation-induced adverse events, categorized according to the CTCAE, were collectively observed in 36 of 147 patients (24.5%). This included 27 patients with CTCAE Grade 1 (20.4%), 3 with Grade 2 (2.0%), 5 with Grade 3 (4.1%), and 1 with Grade 4 (0.7%) toxicity. During the acute phase (within 3 weeks post-SRS), symptoms such as nausea, lethargy, and headache were reported in 11 patients with Grade 1 toxicity. In addition, three patients with Grade 2 toxicity experienced facial numbness and pain 3 months after SRS, which were effectively managed with medication, and one patient with Grade 3 toxicity presented with new-onset generalized seizures, necessitating additional antiseizure medications.

**Figure 2:**
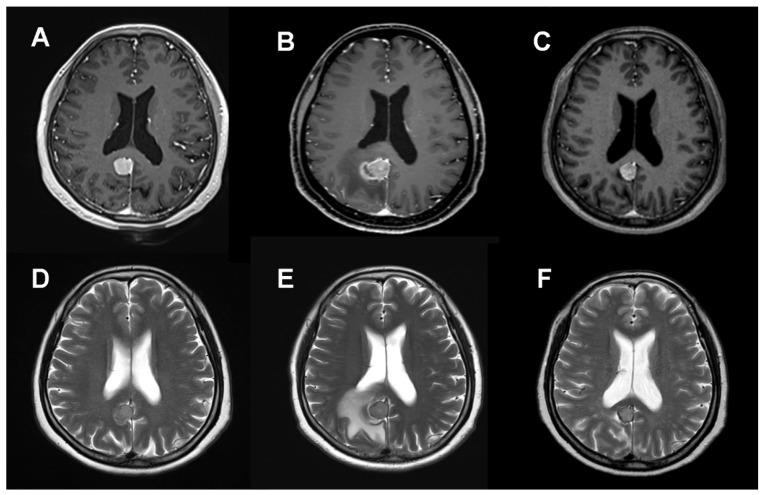
Axial contrast-enhanced T1- and T2-weighted magnetic resonance imaging scans of meningioma at the posterior falx at the time of radiosurgery (**A**,**D**), 6 months later (**B**,**E**), and 3 years later (**C**,**F**). The 6-month follow-up images demonstrate peritumoral edema around the treated meningioma, which was managed with high-dose steroids. Meningioma and peritumoral edema were stabilized 3 years after radiosurgery. Radiosurgery was performed using a tumor margin dose of 15 Gy.

Notably, no instances of clinical or radiological radiation necrosis were identified post-SRS. Radiation-induced PTE directly attributable to SRS was observed in 21 of 164 lesions (12.8%), manifesting approximately 6 months post-treatment (Figure 2). Among these cases, 16 (9.8%) were classified as CTCAE Grade 1–2, and 5 (3.0%) as CTCAE Grade 3–4. Asymptomatic mild edema, which required no active intervention, was observed in 16 patients. In cases of symptomatic PTE (CTCAE 3), four patients were managed with oral (three cases) and intravenous steroids (one case). Only one case necessitated open resection due to uncontrolled seizures associated with abnormal pachymeningeal thickening around the tumor and PTE (CTCAE 4). A comparison of the PTE incidence between the two groups revealed a significantly higher frequency in Group 1 (12 lesions, 19.7%) than in Group 2 (9 lesions, 8.7%) (*p* = 0.039). Furthermore, severe edema (CTCAE Grade 3–4) was more prevalent in Group 1 (6.6%) than in Group 2 (1.0%) (*p* = 0.042). When evaluating the factors related to new-onset or worsened edema after SRS, pre-existing PTE and tumor volume > 10 cm^3^ were significantly associated both in univariate and multivariate analyses. A marginal dose ≥ 14 Gy (Group 1) showed meaningful significance in the univariate analysis but not in the multivariate analysis (Table 5).

## 4. Discussion

The primary objective in managing benign meningiomas is to attain sustained, long-term control, achievable through surgical intervention or radiosurgery. Specifically, within the SRS domain, the administered radiation dose is a pivotal determinant in accomplishing effective local control. Commonly reported SRS doses range from 12 to 18 Gy, meticulously tailored to consider tumor size and its proximity to critical anatomical structures [15,18,19]. Numerous studies have attempted to ascertain the optimal radiation dose for low-grade meningiomas. However, most of these investigations are retrospective, emanating from single-center studies characterized by heterogeneous patient cohorts. Only a few studies have directly compared distinct radiation doses.

Ganz et al. [20] identified an increased risk of treatment failure in cases where the tumor edge doses were <10 Gy, compared with the group receiving doses > 12 Gy, thereby proposing 12 Gy as the minimum threshold for efficacious SRS in meningioma treatment. Conversely, another study demonstrated no discernible advantage in tumor control with marginal doses surpassing 15 Gy compared with doses below this threshold [21]. Similarly, no significant difference was found in the LCR for benign meningiomas at the 5-year mark when the contrasting doses were <16 Gy and >16 Gy, suggesting that higher doses may not uniformly confer additional benefits [22]. In a long-term retrospective study, elevated recurrence rates were reported in patients receiving doses of ≤13.4 Gy, highlighting the intricate balance required in determining an optimal dose that balances efficacy and safety [23]. Pollock et al. [24] corroborated these findings and reported a 10-year LCR of 99.4% with a mean tumor margin dose of 15.8 Gy in an updated study. Collectively, these studies underscore the importance of a personalized approach in radiation therapy, factoring in the minimum effective dose and potential risks associated with higher doses. However, these insights, predominantly derived from single-center investigations, are yet to establish definitive dosing guidelines. Consequently, reliance on recommendations from authoritative bodies such as the Radiation and Oncology Advisory Committee on Radiation Oncology Practice and the National Comprehensive Cancer Network has been advocated, suggesting a dose range of 12–16 Gy [25,26].

At our institution, adhering to the recommended radiation dose for SRS for treating low-grade meningiomas is a consistent practice. However, periods of dose reduction have been implemented, allowing us to compare two distinct cohorts subjected to varying radiation doses.

Patients diagnosed with benign meningiomas generally exhibit a favorable long-term prognosis; however, it is imperative to consider the potential toxicity and delayed effects associated with the treatment itself. The occurrence and nature of toxic effects are contingent on variables such as tumor size and location [4]. In our study, instances of radiation-induced toxicity were observed in 40 of the 147 patients (27.2%) as assessed using the CTCAE. This observed frequency exceeded the overall rate of 8.1% (range: 2.5–28.2) reported in previous meta-analyses [4,13,27]. This discrepancy may be attributed to the heterogeneity in the definition of radiation-induced toxicity and variations in the evaluation tools employed. Our study scrutinized even mild clinical symptoms using CTCAE following SRS, with more clinically symptomatic events (CTCAE grade 3–4) accounting for only 4.8% (7 of 147 patients), consistent with findings from previous investigations.

In addition, the emergence of new-onset or exacerbated PTE constitutes an objective imaging finding that is pivotal in determining treatment outcomes. Previous studies have reported that the incidence of PTE in patients undergoing SRS for meningiomas ranges from 15% to 28% [28,29,30,31,32,33]. Factors such as larger targeted tumor volume, hemispheric tumor location, pre-existing PTE before SRS, and higher marginal dose or maximum dose have been associated with an elevated risk of PTE [28,31,34]. Our results also showed that a large tumor volume > 10 cm^3^ and pre-existing PTE were significantly related to PTE after SRS. Regarding dose prescription, significant association was observed between a marginal dose > 16 Gy and post-SRS PTE [31]. Similarly, a higher frequency of post-SRS complications was reported in cases with a median marginal dose of 17 Gy compared with 14 Gy [10]. Huang et al. demonstrated that a total marginal dose > 14 Gy significantly affected the occurrence of peritumoral edema after SRS, similar to our results [35]. The relative edema indices reach their maximum values at 11 months post-SRS and subsequently decline, with symptom resolution occurring within 24 months in most patients [29,30,34,36]. In the present study, PTE was observed in 21 of 164 lesions (12.8%), with the majority manifesting at 6 months post-treatment. Comparing the overall incidence of PTE between the two groups showed a disparity, with rates of 19.7% in Group 1 and 8.7% in Group 2.

Recently, significant advancements have been made in the radiobiological aspects related to brain tumors, with growing interest in radiosensitizers or radioenhancers to improve the therapeutic efficacy of SRS [37,38,39]. Additionally, ongoing research is exploring immunotherapeutic options as alternatives to steroids for the management of radiation-induced adverse effects [40,41]. These combined efforts indicate the potential expansion of SRS applications by integrating radiobiological and molecular methodologies.

Collectively, these observations imply that the prescription of radiation doses ≥ 14 Gy in SRS treatment for benign meningiomas yields no substantial advantages in tumor control while significantly amplifying the incidence of radiation-induced side effects. Consequently, based on the conclusions drawn from this study, radiation doses < 14 Gy could be considered in SRS treatment for patients with benign meningiomas.

### Limitations of the Present Study

This study was inherently constrained by its single-center, modest sample size and retrospective design. Furthermore, a substantial proportion of the enrolled patients underwent SRS for radiographically presumed benign meningiomas, potentially encompassing higher-grade tumors, thereby introducing a potential confounding variable that may adversely impact study outcomes. Notably, Group 2 patients receiving radiation doses < 14 Gy were treated relatively recently, resulting in a shorter mean follow-up duration than that of Group 1. This temporal discrepancy limits the comparability of outcomes, particularly in long-term follow-up assessments. Another limitation of this study is its exclusive use of participants treated with the TrueBeam system, which makes it challenging to extrapolate the findings to other radiosurgical instruments such as the Gamma Knife or CyberKnife. While there are variations in treatment planning techniques among these devices, the fundamental principles of treatment and the prescribed doses for benign tumors remain largely consistent. Thus, this study holds significance in providing meaningful reference data, rather than absolute values.

Nevertheless, the significance of this study lies in its exclusive consideration of the radiation dose as a variable, with a deliberate effort to mitigate various confounding factors. Future investigations should adopt a structured approach, necessitating large-scale prospective studies with extended follow-up periods to pursue safer and more effective treatments for benign meningiomas.

## 5. Conclusions

SRS is a highly effective therapeutic modality for benign meningiomas and serves as a complementary intervention to open surgery. A critical determinant of SRS efficacy is the administered radiation dose. Higher radiation doses are traditionally correlated with enhanced tumor control; however, they concurrently increase the likelihood of treatment-related complications, underscoring the necessity for a nuanced equilibrium between LCR and complication rates. This study revealed that a radiation dose < 14 Gy did not result in a statistically significant variance in LCR; however, it was associated with a diminished toxicity rate. Therefore, such a dose regimen should be considered in future treatment strategies to balance therapeutic efficacy and safety.

## Figures and Tables

**Table 1:** Clinical characteristics of patients with benign meningiomas.

Characteristics	Number (*n* = 147)
Sex (M/F)	35/112
Mean age in years (range)	61 (37–79)
Clinical presentation	
Asymptomatic	81
Headache	26
Dizziness	18
Visual symptoms	4
Nausea	1
Motor weakness	3
Hearing decline or loss	2
Facial pain or numbness	4
Facial palsy	2
Tremor	3
Seizure	1
Others	2
Neurologic manifestation	
Nonspecific	134
Hemiparesis	4
Dysesthesia	2
Visual decline	1
6th nerve palsy	2
7th nerve palsy	2
8th nerve dysfunction	2
Pathology	
Meningothelial	14
Angiomatous	2
Fibroblastic	1
Microcystic	1
Transitional	4
Mixed	3
No pathological diagnosis	122
Tumor location (164 lesions)	
Skull base	60
Convexity	42
Parasagittal	5
Falcine/tentorium	56
Intraventricular	1

**Table 2:** Treatment parameters according to subgroups.

	Group 1	Group 2	*p* Value
Number of lesions	61	103	
Lesion location (skull base/non-skull base)	24/37	36/67	0.572
Target volume (cm^3^, mean ± SD)	1.75 ± 1.62	1.90 ± 2.24	0.628
Coverage (%, mean ± SD)	99.48 ± 0.69	99.43 ± 0.61	0.653
Conformity index (mean ± SD)	1.76 ± 0.40	1.72 ± 0.51	0.630
Prescription dose (Gy, mean, range)	15 (14–16)	13 (12–13.5)	<0.001
Maximum dose (Gy, mean, range)	17.3 (16.0–20.4)	15.8 (14.2–23.6)	<0.001
Minimum dose (Gy, mean, range)	13.5 (7.5–15.2)	11.5 (5.8–14.4)	<0.001
Mean dose (Gy, mean, range)	16.4 (15.3–19.0)	14.9 (12.8–19.2)	<0.001

**Table 3:** Radiosurgical outcomes and radiation-induced edema.

Outcomes	Total (164 Lesions)	Group 1 (61 Lesions)	Group 2 (103 Lesions)
Mean follow-up duration (months)	47 (range: 12–122)	68 (range: 12–122)	35 (range: 12–119)
Overall tumor control rate	156/164 (95.1%)	57/61 (93.4%)	99/103 (96.1%)
Complete response	0	0	0
Partial response	10 (6.1%)	4 (6.6%)	6 (5.8%)
Minimal response	12 (7.3%)	8 (13.1%)	4 (3.9%)
Stable	134 (81.7%)	45 (73.7%)	89 (86.4%)
Progression	8 (4.9%)	4 (6.6%)	4 (3.9%)
Radiation-induced peritumoral edema	21 (12.8%)	12 (19.7%)	9 (8.7%)
CTCAE 1–2	16 (9.8%)	8 (13.1%)	8 (7.7%)
CTCAE 3–4	5 (3.0%)	4 (6.6%)	1 (1.0%)

CTCAE; Common Terminology Criteria for Adverse Events.

**Table 4:** Factors associated with local tumor control.

	Factor	Univariate	Multivariate		
		*p* Value	HR	95% CI	*p* Value
Local tumor control	Age > 65 years	0.404			
	Female sex	0.449			
	Tumor volume > 10 cm^3^	<0.001	48.651	4.672–506.611	0.001
	Marginal dose ≥ 14 Gy	0.628			
	Location (skull base/non-skull base)	0.728			
	Prior surgery	0.004	7.806	1.735–35.110	0.007

CI, confidence interval.

**Table 5:** Factors associated with post-radiosurgical peritumoral edema.

	Factor	Univariate	Multivariate		
		*p* Value	HR	95% CI	*p* Value
Peritumoral edema	Age > 65 years	0.353			
	Female sex	0.232			
	Tumor volume > 10 cm^3^	0.017	14.242	1.782–113.844	0.012
	Marginal dose ≥ 14 Gy	0.039	2.189	0.894–5.365	0.087
	Location (skull base/non-skull base)	0.200			
	Prior surgery	0.320			
	Pre-existing peritumoral edema	<0.001	12.426	4.616–33.453	<0.001

CI, confidence interval.

## Data Availability

The data presented in this study are available on a specific request from the corresponding author.
